# Protocol for pragmatic randomized clinical trial to evaluate the completion of treatment of latent *Mycobacterium tuberculosis* infection with Isoniazid in the 300 mg formulation

**DOI:** 10.1371/journal.pone.0281638

**Published:** 2023-02-21

**Authors:** João Paulo Cola, Thiago Nascimento do Prado, Bárbara Almeida Campos, Bárbara Juliana Pinheiro Borges, Bárbara Manuella Cardoso Sodré Alves, Sonia Vivian de Jezus, Carolina Maia Martins Sales, Wildo Navegantes de Araújo, Noemia Urruth Leão Tavares, Ethel Leonor Noia Maciel

**Affiliations:** 1 Postgraduate Program in Collective Health, Laboratory of Epidemiology, Universidade Federal do Espírito Santo, Vitória, Espírito Santo, Brazil; 2 Postgraduate Program in Pharmaceutical Sciences, Universidade de Brasília, Brasília, Distrito Federal, Brazil; 3 Postgraduate Program in Tropical Medicine, Universidade de Brasília, Brasília, Distrito Federal, Brazil; The University of Georgia, UNITED STATES

## Abstract

**Introduction:**

It is essential to strengthen the treatment of latent tuberculosis infection (LTBI) to break the chain of transmission. The drug used worldwide for the treatment of LTBI is Isoniazid. A clinical trial conducted in Brazil has demonstrated the bioequivalence of Isoniazid in the 300 mg formulation with 3 tablets in the 100 mg formulation. Further studies are needed to evaluate the completion of treatment with Isoniazid 300 mg single tablet.

**Objective:**

Describing a protocol for a clinical trial to evaluate the completion of treatment of LTBI with the drug Isoniazid in 300 mg tablet formulation compared to the use of Isoniazid in 100 mg tablet formulation.

**Methods:**

This is a pragmatic, multicenter, randomized, open-label clinical trial registered on the Rebec RBR-2wsdt6 platform. Individuals 18 years of age or older with an indication for treatment of LTBI will be included, with only 1 individual per family nucleus. Individuals whose index case of active TB is categorized as retreatment, multidrug-resistant and extremely resistant, individuals transferred from the original center two or more weeks after the onset of treatment, and persons deprived of liberty will be excluded. The study intervention will be the treatment of LTBI with 1 tablet of Isoniazid 300 mg. The control group will receive the treatment of LTBI with 3 tablets of Isoniazid 100 mg. Follow-up will be performed at month 1, month 2 and at the end of treatment. The primary outcome will be completion of treatment.

**Conclusion:**

It is expected that with the treatment with the 300 mg formulation, more patients will complete the treatment based on the complexity index of pharmacotherapy. Our study intends to substantiate theoretical and operational strategies that respond to the demand for incorporation of a new formulation of the drug for the treatment of LTBI in the Unified Health System network.

## Introduction

The “End of TB” strategy has bold targets for controlling the disease: reducing 95% of deaths and 90% in incidence of the disease by 2035 [1]. Strengthening the treatment of latent tuberculosis infection (LTBI) is essential to break the chain of transmission. It is estimated that a quarter of the world’s population is infected with *Mycobacterium tuberculosis* (MTB), but only 5–10% will develop active TB within 2–5 years of exposure to MTB [2, 3]. Although most of this population do not manifest symptoms, the evolution depends on some factors [3–5].

The drug used worldwide for the treatment of LTBI is isoniazid [4, 5]. Isoniazid can reduce the risk of becoming ill with active TB by 60–90%, considering adherence and duration of treatment [4–7]. The World Health Organization (WHO) recommends Isoniazid at a dose of 5 to 10mg / kg / day (with a maximum dose of 300 mg / day) in 180 doses over 6 to 9 months or 270 doses over 9 to 12 months for the treatment of LTBI [5, 8]. A meta-analysis described that the regimen of associated use of Isoniazid with Rifapentine for 3 months (3HP) shows greater completion of treatment compared to the use of Isoniazid for 9 months (9H), however, no significant effects were demonstrated on the outcome of the onset of active TB [9]. Shortening the treatment time of the 3 HP regimen and reducing adverse effects may favor greater completion [9].

In Brazil, Isoniazid is widely used in the 100 mg tablet formulation [5, 8]. A clinical trial conducted in Brazil showed bioequivalence of Isoniazid 300 mg with 3 tablets in the 100 mg formulation [10]. Isoniazid 300 mg has been available in Brazil’s National List of Essential Medicines since 2018 and has been used in the treatment of LTBI in patients with HIV/AIDS. The safety of using the 300 mg formulation makes it possible to expand its use to the entire population with LTBI.

In this sense, it is necessary to advance the studies on the use of Isoniazid 300 mg single tablet. This article aims to describe the protocol of a pragmatic clinical trial and its main hypothesis is that the use of the drug Isoniazid 300 mg presents greater completion of treatment than the use of 3 tablets of 100 mg each.

## Methods

### Study design

**Fig 1:**
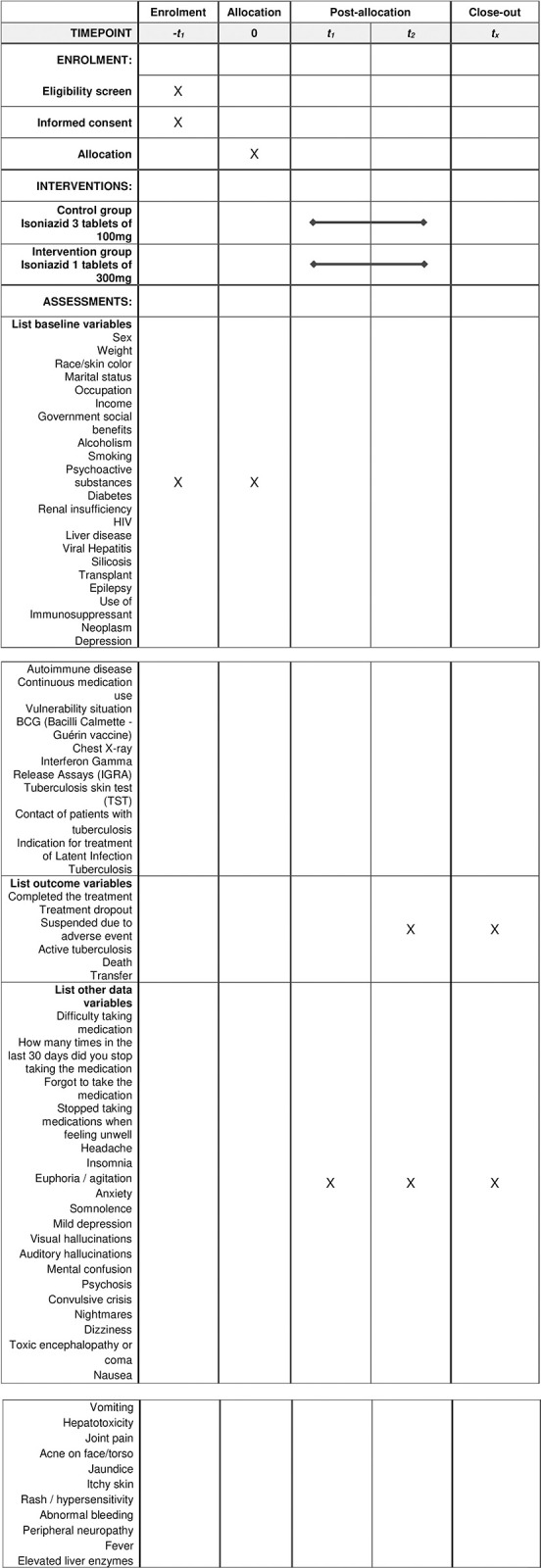
Schedule of enrolment, interventions, and assessments for the protocol for pragmatic randomized clinical trial to evaluate the completion of treatment of latent *Mycobacterium tuberculosis* infection with Isoniazid in the 300 mg formulation, Brazil, from 2019 to 2022.

The protocol outlines a pragmatic, randomized, multicenter, open-label superiority clinical trial to determine the safety, adherence, and completion of treatment of LTBI with the drug Isoniazid in 300 mg tablet formulation (Fig 1). The study will be conducted following the 2010 Consolidated Standards of Reporting Trials guidelines [11].

The study will be conducted in Brazil, and it was designed to include the participation of six centers (Brasília-DF, Curitiba-PR, Belo Horizonte-MG, Fortaleza-CE, Vitória-ES, Serra-ES, Cariacica-ES) selected by convenience in the period from January 2019 to December 2022. The protocol is conducted in Health Units that serve patients with tuberculosis and perform the prescription of treatment of LTBI.

### Selection of individuals

#### Inclusion

People over 18 years of age who are able to express their free and unrestricted will to participate in the study and who are indicated for treatment of LTBI according to the recommendations of the surveillance Protocol for latent *Mycobacterium tuberculosis* infection are eligible, with only one individual per family nucleus [8].

#### Exclusion

Individuals whose index case of active tuberculosis is retreatment, multidrug-resistant and extremely resistant, individuals transferred from the original center after two or more weeks of starting treatment, persons deprived of liberty are excluded from the study.

### Intervention and follow-up

The intervention in the study is treatment of LTBI with INH 300 mg. The individuals will receive 1 tablet of INH 300 mg. The control group will receive the treatment of LTBI with 3 tablets of INH 100 mg.

**Fig 2:**
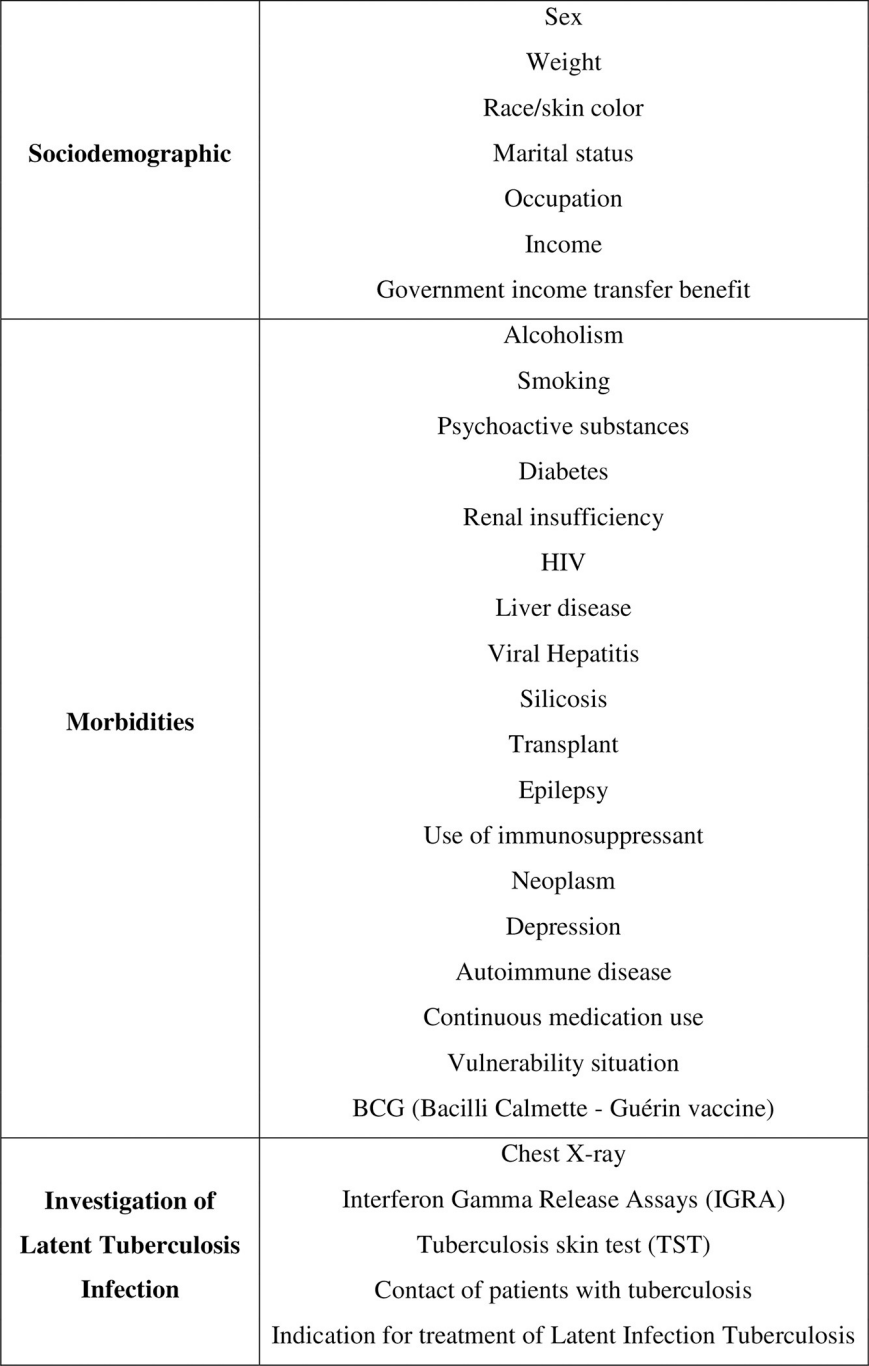
Baseline variables of interest in the pragmatic randomized clinical trial to evaluate the completion of treatment of latent *Mycobacterium tuberculosis* infection with isoniazid in 300 mg formulation, Brazil, from 2019 to 2022.

At baseline, everyone will be oriented by the professional responsible for his/her treatment regarding medicine intake and possible adverse effects. The individuals will be interviewed at baseline of the onset of the treatment using the initial questionnaire with the variables described in Fig 2. Variables were selected based on the forms proposed in the Protocol for surveillance of latent *Mycobacterium tuberculosis* infection, and the pilot study was conducted in November 2018 [8].

**Fig 3:**
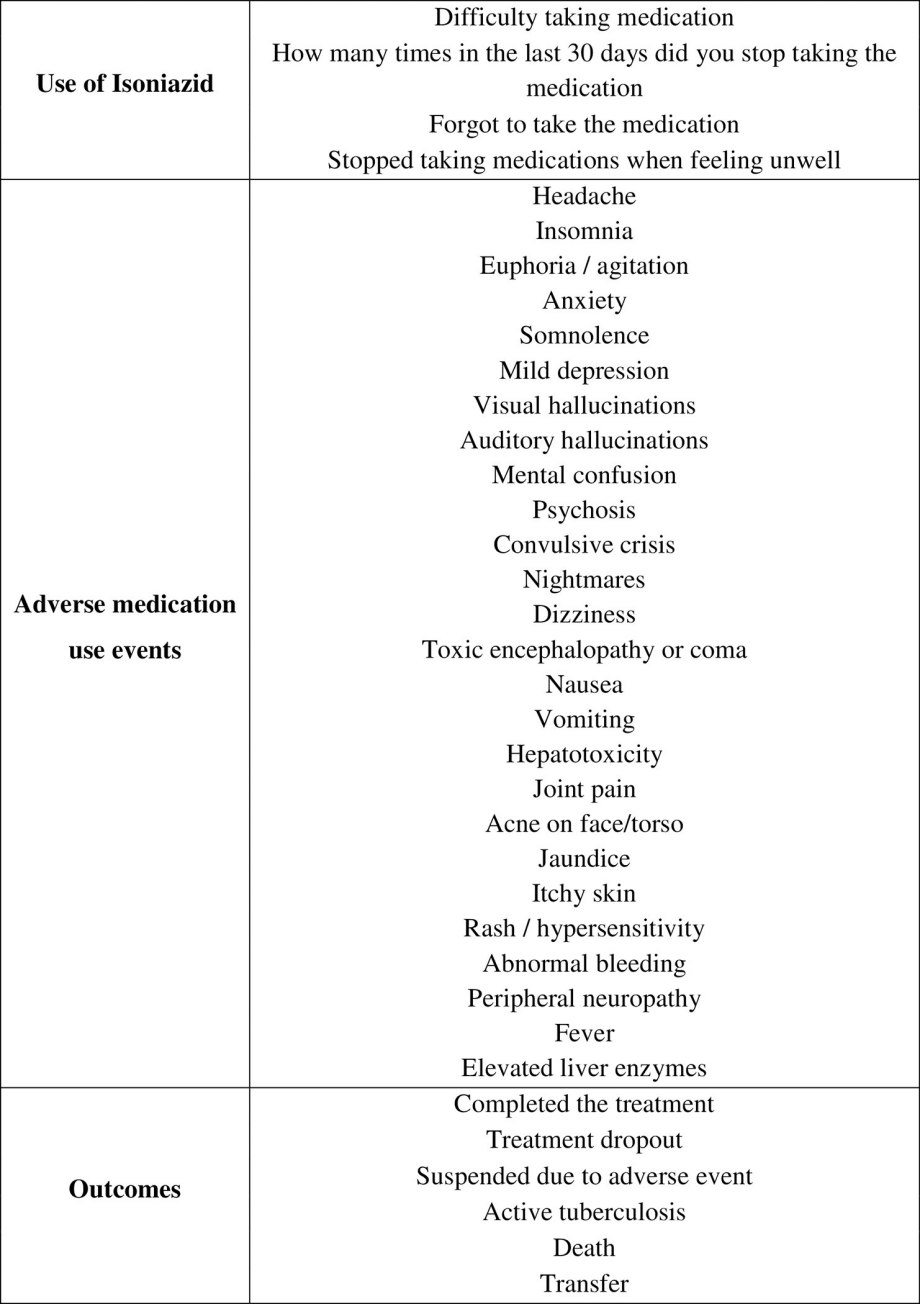
Variables of interest in the follow-up of the pragmatic randomized clinical trial designed to evaluate the completion of treatment of latent *Mycobacterium tuberculosis* infection with isoniazid in 300 mg formulation, Brazil, from 2019 to 2022.

At follow-up, the individuals will be followed up during the period of the LTBI treatment which can last from 6 to 12 months. Data will be collected in accordance with medication use and adverse effects of treatment in month 1, month 2 and at the end of treatment (Fig 3).

### Outcomes

The analytic variable of primary interest will be completion of treatment, determined by the use of 270 doses that can be taken from 9 to 12 months and/or 180 doses that must be taken between 6 and 9 months [8]. The recommendation in Brazil is to carry out the treatment of 270 doses for 9 to 12 months. The choice of the therapeutic regimen of 180 doses can also be used at the discretion of the medical professional accompanying the patient [8]. Individual adherence to the treatment of LTBI will be assessed by clinical interview (self-report) or tablet count.

There is evidence of greater efficacy of treatment with isoniazid for LTBI for individuals who complete at least 80% of the doses [11–20]. Thus, complete treatment will be considered if the individual reported (self-report) taking ≥ 80% of the doses, that is, taking 144 or 216 doses of the 180 or 270 prescribed during the follow-up period, respectively, or tablet count used equal to or greater than 80% of the prescribed doses. The identification of treatment abandonment will be given by individuals who remained three months without medication, consecutive or non-consecutively [8].

The secondary outcomes evaluated in the study will be the identified adverse effects which are: nausea, vomiting, epigastralgia, arthralgia, headache, insomnia, euphoria, agitation, anxiety, drowsiness, acne on face/trunk, itchy skin, visual and auditory hallucination, mental confusion, petechiae, abnormal bleeding, peripheral neuropathy, and jaundice.

### Sample size

The sample size was calculated based on the primary outcome of the completion of treatment of LTBI. Non-completion rates vary between 20 and 40%, with most studies reporting 30% of the treatment abandonment of LTBI [11–14].

For the sample calculation, we used an expec abandonment reduction rate is expected to be around 11%. Abandonment in the intervention group (INH 300 mg) would be 19% and in the control group (INH 100 mg) 30%. With a power of 80% to detect differences, a 5% significance level, using Pearson’s chi-square test, two-sided, about 478 individuals would be needed. Besides using the STATA 14.0 program, a 15% loss was also considered, and the number was corrected to 548 study individuals.

### Randomization and blinding

Individuals will be allocated into two therapeutic regimen groups for the treatment of LTBI. The intervention group will receive INH 300 mg in 1 tablet and the control group will receive INH 100 mg in 3 tablet through a 1:1 randomization process with allocation in blocks of 10 individuals. This strategy ensures the comparison of the intervention and control groups. The study is not blinded to individuals or researchers. The randomization procedure will be performed using the R software and sent in a sealed envelope to each center that participates in the study.

### Recruiting

This is a pragmatic study in which individuals are recruited on a daily basis from routine health care facilities that provide care for individuals with LTBI. Recruitment started on January 2019 and the study termination date was scheduled to December, 2022.

### Data management and analysis plan

Data collected on the instrument will be entered by the participating center into the Research Electronic Data Capture (REDCap) digital electronic case report platform. The coordinating center will audit the data entered periodically to ensure data completion and accuracy. The database will generate a code for each patient to protect the identity of the individuals and confidentiality of sensitive information.

In accordance with Law No. 13709/2018 on Data Protection, persons with direct access to the data will take all necessary precautions to ensure confidentiality [21]. During or at the end of the study, under no circumstances should the names or addresses of patients be exposed. Only the first letter of the subject’s last name and first name will be recorded, accompanied by a research-specific coded number indicating the order of inclusion of the individuals.

In our interim analysis plan, the explanatory variable of primary interest will be the completion of treatment of LTBI. Other variables will be used in the analyses considering their hierarchical relationships and confounding with the primary outcome: demographic and socioeconomic factors such as age, sex, place of residence, year of diagnosis, race/skin color, education; therapeutic regimen, health conditions and comorbidities encompass HIV serology status, diabetes mellitus, alcoholism, viral hepatitis, kidney failure/hemodialysis, neoplasia, silicosis, active smoking, transplant history, epilepsy, prolonged corticosteroid use, illicit drug use, and use of immunosuppressants or TNF- α inhibitors.

We will use the algorithm based on multiple imputations of random forests by chained equations, missForest, to handle the missing data. The random forest test uses bootstrap aggregation of multiple regression trees to reduce the risk of overfitting, combining predictions from many trees to produce less-biased parameter estimates and confidence intervals. This technique allows us to accurately predict individual missing values by imputing continuous and/or categorical data and accounting for complex interactions and non-linear relationships. The imputation analysis will be performed with the program R Project (version 3.3.3; R Foundation for Statistical Computing, Vienna, Austria).

To describe the study population, the rates of categorical variables and mean or median with interquartile range (IQR) of numerical variables will be calculated. For analysis of numerical variables, the Shapiro-Wilk test will be used to test normality. To compare the means with the outcome, Student’s t test will be performed, as well as the Mann-Whitney test for median. For categorical variables, Pearson’s chi-square test will be used to compare variable rates with the outcome.

Poisson regression models with robust variance will be used to examine the association of intervention with treatment completion rates. The dependent variable will be the completeness of the treatment and the independent variable will be the intervention. Fitted models will include possible identified confounders. To assess the quality of the adjustments of each model, the Hosmer and Lemeshow test will be performed after each Poisson regression, those situations in which the test statistic is not significant, when p>0.05, the adjustment will be considered adequate.

Results are presented as risk ratio (RR) with 95% confidence interval (95% CI) that can be interpreted as relative risk of the occurrence of completion of treatment. All analyses will be performed in Stata software version 15.0 (Stata Corp, College Station, TX, USA).

### Adverse event reporting

In case of manifestation of an adverse reaction classified as major, with clinical indication to suspend the administration of INH and/or its substitution, in accordance with the Protocol for surveillance of latent infection by *Mycobacterium tuberculosis* in Brazil, the individuals will be withdrawn from the study and followed up until resolution of the adverse events [8, 22]. His/her withdrawal and follow-up will be recorded on forms specific to the study and notification will be provided to ANVISA’s VigiMed system [23].

### Ethical aspects

The individuals are informed about the study and the possibility of refusing and withdrawing at any time without interfering with their regular treatment. They are invited to participate in the investigation and give signing the informed consent form to be included in the study.

The study protocol was reviewed and approved by the Research Ethics Committee of the Health Sciences Center of the Federal University of Espírito Santo (CEP/CCS-UFES) through Opinion n. 2.764.103/2018 and by the National Research Ethics Committee (CONEP) under number 88226218.0.1001.5060. The protocol is registered in the Brazilian Registry of Clinical Trials (REBEC) under code RBR-2wsdt6 (http://ensaiosclinicos.gov.br/rg/RBR-2wsdt6/).

## Discussion

Pragmatic trials, in principle, tend to establish a scientific basis for decision making in situations most commonly found in routine health care practice. From this perspective, the study sample is based on the enrollment of individuals who are representative of the individuals seen in the routine of care services, favoring an evaluation of the intervention in the real scenario where it will occur [24].

The study is carried out in health care facilities that provide care to people with tuberculosis and LTBI, distributed in seven centers in several regions of Brazil. This scenario allows not only to evaluate the safety of INH 300 mg, but also its applicability to the routine of health care services, the effect on the use of the drug, and the completion of treatment.

To ensure validation of the pragmatic trial, the study uses a real-time data entry platform, and the coordinating center monitors the randomization, recruitment, and follow-up of individuals enrolled in the study on a weekly basis [25]. Recruitment of the calculated study sample ensures extrapolation and external validation [25, 26]. Conducting the trial in the context of the COVID-19 pandemic in Brazil may make it difficult to recruit individuals since the care services for people with tuberculosis have been suffering from the transfer of health professionals to work directly with the pandemic [27].

Unblinding may introduce bias into the study, so that it may induce responses from the subject that are different from what has actually occurred and a different evaluation from the evaluator/prescriber of the medication. However, it is difficult to blind the study because the intervention is visually different from the control, with 1 tablet in the intervention group and 3 tablets in the control group. Besides, the intervention depends on medical prescription, which makes it impossible to blind the evaluator/prescriber. In order to minimize the above, data analysis will be performed by a blinded researcher.

## Conclusion

At the end of the study, data will provide elements to answer whether isoniazid in the 300 mg formulation reduces the non-completion of treatment and whether it is a safe drug for use in patients with LTBI. Thus, we aim to design theoretical and operational strategies that will respond to the demand for incorporation of a new formulation of the drug for treatment of LTBI in the Unified Health System network.

## Supporting information

S1 Checklist CONSORT 2010 checklist of information to include when reporting a randomised trial. (DOCX)

S2 Checklist SPIRIT 2013 checklist. (DOCX)

S1 File ReBEC. (PDF)

S2 File The protocol approved by the ethics committee. (DOCX)
