# Supplementary material for: Protocol for pragmatic randomized clinical trial to evaluate the completion of treatment of latent Mycobacterium tuberculosis infection with Isoniazid in the 300 mg formulation
Source: PLoS One. 2023 Feb 21;18(2):e0281638. doi: 10.1371/journal.pone.0281638 (PMC9942980; doi:10.1371/journal.pone.0281638)
Supplement: S1 File — (PDF) [file pone.0281638.s003.pdf]

RBR-2wsdt6 Evaluation of Isoniazide 300mg for Latent Tuberculosis Treatment

Data de registro: 10/09/2018 (dd/mm/yyyy)

Última data de aprovação: 10/09/2018 (dd/mm/yyyy)

Tipo de estudo:

Intervenções

Título cientíkco:

en
Evaluation of implementation of 300mg Isoniazide for the Treatment of Latent Infection of Tuberculosis in Brazil

pt-br
Avaliação da implementação da Isoniazida 300mg para o Tratamento da Infecção Latente da Tuberculose no Brasil

Identikcação do ensaio

- **Número do UTN:** U1111-1215-3100
- **Título público:**

  en
  Evaluation of Isoniazide 300mg for Latent Tuberculosis Treatment

  pt-br
  Avaliação da isoniazida 300mg para o tratamento da tuberculose latente

- **Acrônimo cientíkco:**
- **Acrônimo público:**
- **Identikcadores secundários:**
  - **88226218.0.1001.5060**
    Orgão emissor: Plataforma Brasil
  - **2.764.103**
    Orgão emissor: Comitê de Ética em Pesquisa do Centro de Ciências da Saúde da Universidade Federal do Espírito Santo

Patrocinadores

- **Patrocinador primário:** Universidade Federal do Espirito Santo
- **Patrocinador secundário:**
  - **Instituição:** Universidade Federal do Espirito Santo
- **Fontes de apoio knanceiro ou material:**
  - **Instituição:** Programa Nacional de Controle da Tuberculose

Condições de saúde

- **Condições de Saúde:**

en
Individuals diagnosed with latent tuberculosis. LATENT TUBERCULOSIS; Chemoprevention

pt-br
Indivíduos diagnosticados com tuberculose latente. Descritores: Tuberculose Latente;Quimioprevenção

- **Descritores gerais para condições de saúde:**

en
C23 Pathological conditions, signs and symptoms

pt-br
C23 Condições patológicas, sinais e sintomas

es
C23 Condiciones patológicas, signos y síntomas

- **Descritores especíkcos para condições de saúde:**

Intervenções

- **Intervenções:**

en
546 individuals with a diagnosis of latent tuberculosis will participate in the study. Being 273 in the experimental group and 273 in the control group. As described below: Experimental group: take one (1) 300mg tablet for the treatment of latent tuberculosis infection from 9 to 12 months (oral medication, Isoniazid). 273 individuals will be invited 273 individuals with a diagnosis of latent tuberculosis will be invited. Control group: It will take 3 (three) 100mg for the treatment of latent tuberculosis infection from 9 to 12 months (oral medication, Isoniazid). 273 individuals will be invited 273 individuals with a diagnosis of latent tuberculosis will be invited.

pt-br
Participarão do estudo 546 indivíduos com diagnóstico de tuberculose latente. Sendo 273 no grupo experimental e 273 no grupo controle.Conforme descrito abaixo: Grupo experimental: tomará 1 (um)comprimido de 300mg (via oral) para o tratamento da infecção latente da tuberculose de 9 a 12 meses (isoniazida). Participarão 273 indivíduos com diagnóstico de tuberculose latente. Grupo controle: Tomará 3 (três) comprimidos de 100mg (via oral) para o tratamento da infecção latente da tuberculose de 9 a 12 meses (isoniazida). Participarão 273 indivíduos com diagnóstico de tuberculose latente.

- **Descritores para as intervenções:**

en
D02.442.436 Isoniazid

pt-br
D02.442.436 Isoniazida

es
D02.442.436 Isoniazida

Recrutamento

- **Situação de recrutamento:** Ainda não recrutando
- **Países de recrutamento**
  - Brasil
- **Data prevista do primeiro recrutamento:** 01/10/2018 (dd/mm/yyyy)
- **Data prevista do último recrutamento:** 01/04/2019 (dd/mm/yyyy)

Tamanho da amostra alvo:	Gênero para inclusão:	Idade mínima para inclusão:	Idade máxima para inclusão:
546	-	18 Y	80 Y

- **Critérios de inclusão:**

en
All individuals over 18 years of age with indication for ILTB treatment

pt-br
Todos os indivíduos maiores de 18 anos com indicação de tratamento da

- **Critérios de exclusão:**

en	pt-br
Individuals whose case index is a multiresistant and extremely resistant case, as well as those individuals who were transferred from the original center after two or more weeks of treatment initiation beyond the individuals in the prison system will be excluded.In addition, people over 80 years old will be excluded.	Serão excluídos os indivíduos cujo caso índice for caso de multirresistente e extremamente resistente, bem como aqueles indivíduos que foram transferidos do centro original depois de duas ou mais semanas do início do tratamento além dos indivíduos do sistema prisional. Indivíduos maiores de 80 anos

Tipo de estudo

- **Desenho de estudo:**

en	pt-br
Clinical trial of treatment, randomized, parallel, open, with two arms.	Ensaio clínico de tratamento, randomizado, paralelo, aberto, com dois braços.

Programa de acesso expandido	Enfoque do estudo	Desenho da intervenção	Número de braços	Tipo de mascaramento	Tipo de alocação	Fase do estudo
	Tratamento	Paralelo	2	Aberto	Randomizado controlado	N/A

Desfechos

- **Desfechos primários:**

en	pt-br
Difference in number of patients who adhered to the treatment of latent tuberculosis, verified by the follow-up of the patients and the finding of at least 5% increase in the success rate to the treatment with the intervention.	diferença no número de pacientes que aderiram ao tratamento da tuberculose latente, verificado pelo acompanhamento dos pacientes e com a constatação de pelo menos 5% de aumento na taxa de sucesso ao tratamento com a intervenção.

- **Desfechos secundários:**

en	pt-br
Secondary outcomes are not expected	Não são esperados desfechos secundários

Contatos

- **Contatos para questões públicas**
  - **Nome completo:** Carolina Maia Martins Sales
  -
    - **Endereço:** Avenida Fernando Ferrari, 514, goiabeiras
    - **Cidade:** Vitoria / Brazil
    - **CEP:** 29075-910
  - **Fone:** +55-027-33357013
  - **Email:** carolnurse@hotmail.com
  - **Akliação:** Universidade Federal do Espirito Santo

- **Contatos para questões cientíkcas**
  - **Nome completo:** Ethel Leonor Noia Maciel

- - **Endereço:** Avenida Fernando Ferrari, 514, Goiabeiras
    - **Cidade:** Vitoria / Brazil
    - **CEP:** 29075-910
  - **Fone:** +55-027-33357013
  - **Email:** ethel.maciel@gmail.com
  - **Akliação:** Universidade Federal do Espirito Santo

- **Contatos para informação sobre os centros de pesquisa**
  - **Nome completo:** Ethel Leonor Noia Maciel
  - - **Endereço:** Avenida Fernando Ferrari, 514, Goiabeiras
    - **Cidade:** Vitoria / Brazil
    - **CEP:** 29075-910
  - **Fone:** +55-027-33357013
  - **Email:** ethel.maciel@gmail.com
  - **Akliação:** Universidade Federal do Espirito Santo
  - **Nome completo:** Thiago Nascimento do Prado
  - - **Endereço:** Avenida Fernando Ferrari, 514, Goiabeiras
    - **Cidade:** Vitoria / Brazil
    - **CEP:** 29075-910
  - **Fone:** +55-027-33357013
  - **Email:** thiagonprado@gmail.com
  - **Akliação:** Universidade Federal do Espirito Santo
