# Supplementary material for: Protocol for pragmatic randomized clinical trial to evaluate the completion of treatment of latent Mycobacterium tuberculosis infection with Isoniazid in the 300 mg formulation
Source: PLoS One. 2023 Feb 21;18(2):e0281638. doi: 10.1371/journal.pone.0281638 (PMC9942980; doi:10.1371/journal.pone.0281638)
Supplement: S2 File — (DOCX) [file pone.0281638.s004.docx]

**MINISTÉRIO DA SAÚDE**

**PROGRAMA NACIONAL DE CONTROLE DA TUBERCULOSE**

**UNIVERSIDADE FEDERAL DO ESPÍRITO SANTO**

**LABORATÓRIO DE EPIDEMIOLOGIA**

**EVALUATION OF IMPLEMENTATION OF ISONIAZIDE 300MG FOR THE TREATMENT OF LATENT TUBERCULOSIS INFECTION**

**VITÓRIA 2018**

**SUMÁRIO**

[**INTRODUCTION 3**](#_heading=h.gjdgxs)

[**OBJECTIVE 6**](#_heading=h.30j0zll)

[**METHODS 7**](#_heading=h.1fob9te)

[**SCHEDULE 20**](#_heading=h.3znysh7)

[**BUDGET 21**](#_heading=h.2et92p0)

[**EXPECTED RESULTS 22**](#_heading=h.tyjcwt)

[**BIBLIOGRAPHIC REFERENCES 23**](#_heading=h.3dy6vkm)

[**APPENDICES 27**](#_heading=h.1t3h5sf)

# INTRODUCTION

The World Health Organization (WHO) defines latent Mycobacterium tuberculosis infection (LTBI) as a state of persistent immune response to stimulation by Mycobacterium tuberculosis antigens without evidence of clinical manifestations of active tuberculosis (TB). It is estimated that a quarter of the world population is infected by this bacterium. Despite the absence of symptoms, there is a risk of these patients developing active TB, especially in the first two years after the primary infection worsened in the case of immunosuppression. Activation occurs in 5-10% of cases (LEITE-JÚNIOR, 2017).

Thus, the evaluation of contacts of TB cases is of relevant importance for the screening of LTBI, which requires proper diagnosis and treatment, with the aim of preventing the development of active TB in the future, also interrupting the disease transmission cycle. In Brazil, the National Tuberculosis Control Program/Ministry of Health (PNCT) considers any person who lives in the same environment as the index case as contact at the time of diagnosis of TB. Contacts under the age of five, people with HIV and people with high-risk conditions should be considered a priority in the process of evaluating contacts and treating LTBI (BRASIL, 2017).

The drug Isoniazid is used worldwide for the treatment of LTBI and reduces the risk of illness by 60 to 90%, depending on adherence and duration of treatment (WHO, 2015). As recommended by the Health Surveillance Guide (2017), the current regimen indicated and available for the treatment of LTBI is with the presentation of Isoniazid 100mg in a dosage of 5 to 10 mg/kg/day (maximum dose of 300mg/day) in 270 doses that should be taken from 9 to 12 months (BRASIL, 2017). However, from 2018, Isoniazid 300mg will also be made available to the health network, which should be incorporated by the services. In other words, there will be two presentations of Isoniazid (100mg and 300mg) in the units studied to be used in the treatment of LTBI. Studies carried out by FIOCRUZ indicate that the two formulations have bioequivalence (Daher et al, 2015).

The two presentations of Isoniazid (100mg and 300mg) will be available to be used as needed depending on the weight range of adults and children. Since overdose of Isoniazid can lead to severe drug hepatitis and death, correct use is imperative to ensure that people undergoing treatment for LTBI receive the correct dose of Isoniazid.

Thus, the triad for the correct use of a medication composed of: prescription, dispensing and ingestion needs to be monitored and questions related to the professionals who carry out the prescription, to those who participate in the dispensing process, which differs in each service, and also, questions related to drug intake by the patient bring together a new configuration in Tuberculosis programs and will therefore be the object of this study, as described in Figure 1.
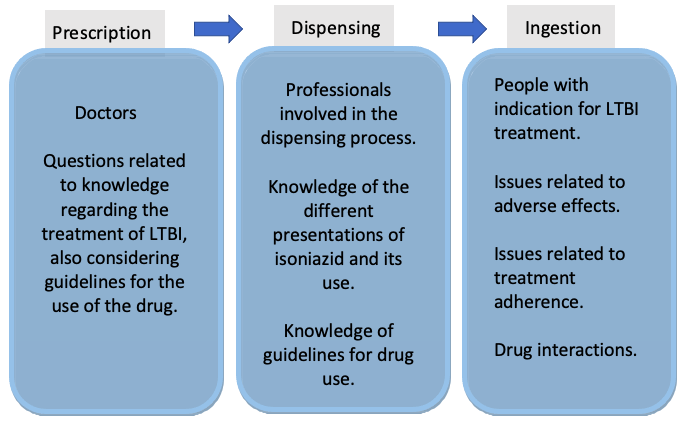


Figure 1 - Processes related to the triad of evaluation of new dosage

Thus, with the development of the present study, it is intended to understand the process of implementation of the presentation of Isoniazid 300mg for the treatment of LTBI in some health units in different regions of the country in order to identify potential risks in the process of using the drug. medication and ensure patient safety.

To carry out this study, three steps will be adopted that may occur simultaneously. The first stage will be a pragmatic clinical trial with patients LTBI diagnosed in health facilities in Brasília, Curitiba, Florianópolis, Ribeirão Preto - SP and Vitória, which will evaluate the prescription, dispensing and ingestion of the two dosages provided by the PNCT. The second stage will be a cross-sectional study using the 'Knowledge, attitude and practices' methodology to verify compliance in prescribing and dispensing. And the third stage will be a qualitative study with health professionals to analyze the speeches of professionals who prescribe the drug and professionals who dispense the new dosage of 300mg. In this sense, the evaluation of this new presentation will consist of stages that complement each other, as shown in Figure 2:


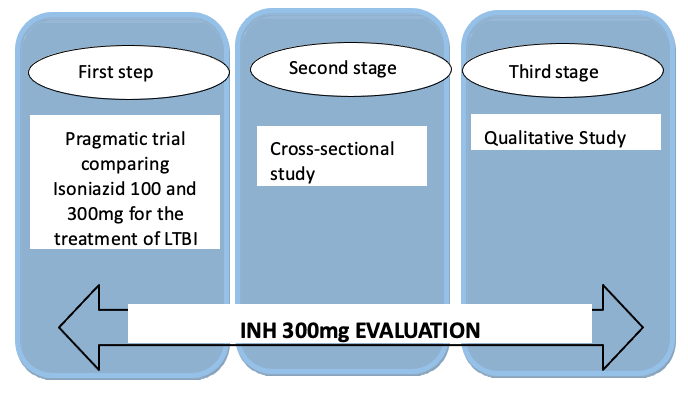


Figure 2. Steps for the Evaluation of Implementation of Isoniazid 300mg for the treatment of LTBI

# OBJECTIVE

• To analyze the occurrence of serious or unexpected adverse reactions resulting from the use of Isoniazid 300mg by the patient for the treatment of latent tuberculosis infection.

• To analyze the patient's use of Isoniazid 300mg for the treatment of latent tuberculosis infection.

• To analyze the information strategies made available to health services on the new presentation of Isoniazid 300mg for the treatment of latent tuberculosis infection.

• To analyze compliance in the prescription of Isoniazid 300mg for the treatment of latent tuberculosis infection through the Knowledge, Attitudes and Practices (CAP) strategy.

•To analyze compliance in the dispensing of Isoniazid 300mg for the treatment of latent tuberculosis infection through the Knowledge, Attitudes and Practices (CAP) strategy.

• To determine the plasma levels of patients undergoing treatment with Isoniazid 100 and 300mg

• To analyze the social representations of health professionals from the basic unit on the use of Isoniazid 300mg for the treatment of latent tuberculosis infection.

• To know the meanings of health professionals at the basic unit about the use of Isoniazid 300mg for the treatment of latent tuberculosis infection.

• To identify the role of health professionals from the basic unit in relation to patients using Isoniazid 300mg for the treatment of latent tuberculosis infection.

• To interpret how health professionals at the basic unit relate their conceptions to the prescription of Isoniazid 300mg for the treatment of latent tuberculosis infection.

• To develop and analyze the use of a computerized system for mobile devices that helps health professionals in the diagnosis and treatment process, as well as to assist in the prevention and care of people with latent Tuberculosis, evaluating its use in these health services to integrate follow-up actions as well as evaluating the degree of solvability of these actions from the system.

# METHODS

STUDY STEPS

First step

Research sites and Study design

This step will be carried out in Brasília, Curitiba, Santa Catarina, Ribeirão Preto, Espírito Santo in the form of a pragmatic, open-label, randomized clinical trial in health units that provide care to patients with Tuberculosis and whether or not they have a Tuberculosis Control Program (PCT acronym in Portuguese). ). The health facilities that prescribe LTBI treatment will be chosen. From June 2018 to March 2019, people over 18 years of age with indication for LTBI treatment will be invited to participate in this study.

After signing the informed consent term, the contacts will be randomized to receive Isoniazid 100mg or 300mg and will be followed up until the end of treatment.

The primary outcome will be the success rate of LTBI treatment in the new 300mg formulation represented by the proportion of subjects with 270 doses of isoniazid between 9 to 12 months.

Secondary outcomes will be evaluated by comparing adverse effects in the two groups (Isoniazid 100mg or 300mg).

**Phase 1- Baseline- before the introduction of Isoniazid 300mg**

Each participant who will receive treatment for LTBI will be guided by the professional responsible for their treatment in relation to taking the medication and possible adverse effects. A medication intake diary containing information about the time of medication intake and the presence of any signs or symptoms of adverse effects will be delivered to the participants through a cell phone application that will be developed for the study. In addition to the diary, this case will be entered into the latent tuberculosis infection treatment reporting system at each research site.

**Phase 2- Follow-up**

Study subjects will be interviewed by the researchers regarding medication intake and adverse effects at the following study periods: week 2, first month, second month, and end of treatment.

Thus, 2 forms will be built, one for diagnosis and another for follow-up (Appendix 1 and 2), based on the forms proposed by the PNCT for the follow-up of these people, already in use in other capitals. The follow-up will also be entered into the notification system for the treatment of latent tuberculosis infection in force in each municipality. The forms will include sociodemographic and clinical information regarding initiation, follow-up and outcome of LTBI treatment. The data can be collected independently in the presence of a data network, whether on cell phones, tablets, notebooks or desktops, if necessary, the data can also be collected in physical media.

In secondary outcomes, in addition, new technologies are being proposed so that the treatment of LTBI can be monitored by health services and so that the health education process, especially applied to TB, can be more easily achieved through an application.

**SAMPLE SIZE CALCULATION**

For the sample size calculation, the parameters of the primary outcome of adherence to LTBI treatment were used. Non-adherence rates vary between studies from 40 to 20%, with most studies being situated between more than 30% abandonment of LTBI treatment. For the sample calculation, we used an expected dropout rate reduction of around 11%. That is, the expected rate of abandonment of the LTBI treatment expected in the 300mg group would be 19% and in the 100mg group, 30%. With a power of 80% to detect differences and a significance level of 5%, it is estimated that 474 subjects are needed in each group. The STATA 15.0 program was used and a loss of 15% was also considered, this number being corrected for 546 study subjects.

**SELECTION OF HEALTH FACILITIES**

The health facilities will be selected among primary health care facilities, Family Health Strategy facilities and Reference Centers for the treatment of TB. All health facilities in the study areas will be identified. Your selection will be made according to the number of individuals who underwent treatment for LTBI in the previous year in each capital.

**SELECTION OF INDIVIDUALS AND RANDOMIZATION**

All individuals over 18 years of age with an indication for LTBI treatment between June 01, 2018 and March 30, 2019 at one of the centers selected for the study will be eligible. Individuals whose index case is retreatment, multidrug-resistant and extremely resistant will be excluded, as well as those individuals who were transferred from the original center after two or more weeks of starting treatment in addition to individuals from the prison system.

186 subjects will be included in Espírito Santo and 90 in each of the centers of the

study.

Study subjects will be randomized through a random number worksheet that will contain the treatment regimen in two study groups: Intervention Group: Isonizazide in the 300mg formulation and Control Group: Isonizazide in the 100mg formulation. Randomization will be by block. A randomization list with a random sequence of blocks of participants will be generated for each study center. This strategy ensures that the intervention group and the control group are balanced in terms of the number of participants. To ensure allocation confidentiality, sealed envelopes with the randomization sequence generated using the R software will be sent to each center during the study.

**ANALYSIS PLAN**

The explanatory variable of primary interest will be the participant's adherence to LTBI treatment. Other explanatory variables will be used in the analysis models, respecting possible hierarchy and confounding relationships: (1) Demographic and socioeconomic factors: age, sex, place of residence, state capital (yes/no), year of diagnosis, ethnicity or skin color (white, black, brown, yellow and indigenous), formal education (number of school years completed); (2) Pre-existing medical conditions and comorbidities: HIV serology, diabetes, alcoholism and mental illness; (3) Variables associated with infection or adverse effects of LTBI treatment.

LTBI treatment success and dropout rates will be calculated for each study group. Student's t test will be used to compare means and Pearson's chi-square test will be used to compare proportions and the variables associated with the outcome of interest (p < 0.20) will be included in the multivariate regression model for adjustment. Results are presented in relative risk (RR) with a 95% confidence interval (95% CI). Ideally, all subjects included in the study should complete it following the initial protocol. This situation almost never happens, for different reasons. In general, there are losses, non-adherence to treatments and other deviations from the protocol. For the analysis of the results, two basic populations will be defined: the first is defined by the “intention-to-treat” (ITT) which will include all patients regardless of protocol violations and loss to follow-up; the second, referred to as “per-protocol” (PP), will be a subset of the first, including only patients who completed treatment according to the protocol criteria, excluding all violators. These two analyzes are important, as a significant violation of the criteria established in the protocol (many losses, switching or poor adherence to treatment, and/or other deviations) in the ITT analysis may include a no-difference bias resulting in a lower treatment effect and greater risk of falsely declaring non-inferiority. On the other hand, the PP analysis, in these adverse situations, may include a difference bias, resulting in a greater effect of the treatments and a greater risk of falsely declaring superiority. Therefore, ITT analyzes are conservative for superiority while PP are conservative for non-inferiority. We will therefore present both and the CONSORT Statement will also be used throughout the report (Schulz, Altman and Moher, 2010). All analyzes will be performed in Stata, version 15.0.

**Subanalysis in the Randomized Trial**

A plasma monitoring study will be conducted on a subsample of the pragmatic clinical trial on LTBI randomly selected for the 100mg and 300mg drug presentation treatment arms of isoniazid from the Federal District sites. The subsample size will comprise 36 subjects (18 per arm of the pragmatic clinical trial).

The health units in which research participants will be invited to participate in the Federal District will be: Health Center of the Structural Administrative Region (1); Day Hospital 509 south (2); Pharmacy- School of the University Hospital of UnB (3). The first health unit is characterized by being a primary care unit located in a city of approximately 39,015 inhabitants (PDAD, 20151), the second being a specialized center both in the care network of the Health Department of the Federal District.

Brachial blood samples will be collected with a tube for serology in order to separate the total plasma in a centrifuge at 2500 rpm for 10 minutes and stored in a 1.5 mL Eppendorf tube at -80o C until the moment of measurement in the chromatography device. high performance liquid (HPLC). The samples will be sent for conservation at -80oC to the Biobank of the Nucleus of Tropical Medicine of the Faculty of Medicine of the University of Brasília (UnB) and later sent to the Toxicology Laboratory of the Department of Pharmacy of the Faculty of Health Sciences of the UnB.

Each research participant (n=36) will be invited, after reading, clarifying and signing the informed consent, to donate a blood sample at three different times of treatment (30 days of treatment; 3rd month of treatment; 6th month (at the end of ) treatment. Each plasma sample will be treated with solvent solution and filtered to prepare the specimen before performing the plasma isoniazid dosage. It will be launched in the HPLC in order to identify its chromatometric curve compared to the available isoniazid standard. In this way, it will be possible to identify the plasma level of isoniazid of participants with LTBI randomly allocated in the 100mg or 300mg arms of LTBI treatment with isoniazid in some possible subgroups: (i) patients with adequate adherence and use correctly foreseen by the Ministry da Saúde2; (ii) underdosage (those who, even with a 300mg prescription, can make a mistake and take a 100mg tablet thinking they are is receiving 300mg due to incorrect understanding or dispensing in a pharmacological presentation below the prescribed 300mg), (iii) overdose (participants who, due to a mistake in the participant's understanding, when using three 300mg tablets when it should have been 100mg); Possible drug interactions including those who are using other drugs, with any unexpected drug interactions; (iv) patients who did not correctly adhere to what was recommended and what was prescribed and informed by the prescriber even though the pharmacological presentation had been correctly dispensed.

Second stage

Study Scenario and Design

A cross-sectional study on knowledge, attitudes and practices (KAP) will be conducted. It is known that the triad of knowledge, attitudes and practices in combination regulate aspects of life in human societies. These three components can be defined as follows: Knowledge is the ability to acquire, retain and use information; a mixture of understanding, experience, insight and skill; Attitudes refer to inclinations to react in a certain way to certain situations; seeing and interpreting events according to certain predispositions; or organize opinions into coherent and interrelated structures; and Practices mean the application of rules and knowledge that lead to action. (Kaliyaperumal K., 2004). This methodology will be used in relation to prescribing and dispensing LTBI treatment, with a sample of primary care health professionals and reference units in Brasília, Curitiba, Santa Catarina, Ribeirão Preto and Espirito Santo.

**Participants and sample size**

All those involved will be invited to participate in prescribing, dispensing and monitoring LTBI treatment in primary care health units and reference units included in stage 1 in Brasília, Curitiba, Florianópolis, Ribeirão Preto and Vitória.

For the sample calculation, the STATA 15.0** program was used. A 70% hit ratio was considered, a power of 80% to detect differences and a significance level of 5%. Thus, it is estimated that 47 subjects are needed. As this is a cross-sectional study, the study design effect of 1.2 was applied. Therefore, 56 subjects will be needed.

**Data collect**

A single individual, self-administered, standardized and semi-structured questionnaire will be used for data collection. This questionnaire will contain questions with socio-demographic characteristics (sex, age, education, profession, family income, time since graduation, etc.) when, workload; if you received training on tuberculosis control, which type, when, workload; if you treat patients with tuberculosis or at least with respiratory symptoms; time in weeks of the last tuberculosis case treated; If you treat patients with LTBI, etc.). Included in Appendix 03

Data collection at all five study centers will be conducted prior to the qualitative focus group study to be conducted in Brasília.

**Assessment of knowledge, attitudes and practices**

We will establish as "knowledge about LTBI" for this study, the respondent who correctly answers at least five of the ten questions described below:

- Do patients with latent tuberculosis infection (LTBI) also have symptoms of pulmonary tuberculosis? Respondents are expected to respond negatively to this question.

- How would you make the laboratory diagnosis of LTBI? The interviewee is expected to answer: "mainly using the tuberculin skin test" and others such as IGRA mainly in patients vaccinated with BCG, in addition to the x-ray.

- How is the tuberculin skin test performed? The respondent is expected to respond: "(The antigen) is applied (0.1mL) intradermally (in the middle third) on the forearm)"

- How long does it take to get the tuberculin skin test result? Respondent is expected to respond: "(Preferably) within 48 to 72 hours of application"

- Would you answer that adults with a tuberculin skin test with a result above 5mm, but normal x-ray, should undergo treatment for LTBI? The respondent is expected to answer "Yes".

- Should children under 10 years of age, contact with a tuberculosis case, receive treatment for LTBI with a tuberculin skin test result > 10mm if vaccinated less than two years ago? The respondent is expected to answer: "Yes (they are even a priority)"

- Should the treatment for LTBI be the responsibility of the primary care service? Respondent is expected to answer "Yes"

- Is the treatment for LTBI conducted using isoniazid or rifampicin? Respondent is expected to answer "isoniazid"

- What is the maximum amount that a patient undergoing LTBI treatment should receive per day of the indicated drug? The respondent is expected to answer "300 mg/day".

- How long is the treatment for LTBI recommended? The respondent is expected to answer "six to nine months"

- In which situations is LTBI treatment recommended for people living with HIV/AIDS? The interviewee is expected to answer “Normal X-ray and PT >= 5mm, contact of a bacilliferous patient independent of PT and PT < 5mm and with a documentary record of having had PT >= and not undergoing chemoprophylaxis; radiological scar of TB without treatment record.

The 2011 Manual of Recommendations for Tuberculosis Control in Brazil (BRASIL, 20113) was used as the standard text for the preparation of the questions. Attitudes and practices will also be measured in an attached questionnaire.

**Data analysis:**

The variables will be summarized using measures of frequency, and of central tendency and dispersion, being those that concern knowledge, attitudes and practices in the face of LTBI care. Categorical covariates will be tested using Pearson's chi-square test (or Fischer's Exact test) and continuous ones with Student's T test (or Wilcoxon) will be tested as factors associated with knowledge about LTBI. Variables tested in the bivariate analysis with p value < 0.20 will be taken to the multivariate analysis (logistic regression). In the end, it is expected to assume as factors associated with the lack of knowledge about LTBI those variables that, at the end of the multivariate analysis, present a value of p <0.05. Data will be collected through an electronic questionnaire with pre-coded and open questions, previously tested in a group of subjects similar to those included in the research to confirm the adequacy of the instrument, aiming at the quality of the information obtained.

Textual responses will be coded. The variables will be grouped as follows: adequate or sufficient and insufficient knowledge.

In the outcomes in relation to CAP, the variables will be grouped as follows: adequate or sufficient knowledge regarding the adequacy of the prescription in both doses and inadequate or insufficient knowledge, when knowing the adequacy of only one of the doses. The attitude will be considered adequate when it agrees with the indication of treatment for all the nominees and inappropriate when it does not agree or does not carry out the indication. Regarding the practice of indicating treatment, it will be divided into two groups: those that indicate treatment for LTBI (group 1) and those that do not (group 2).

Similarly, professionals who participate in the dispensing process at baseline will be evaluated. The variables will be grouped as follows: adequate or sufficient knowledge, when the professional is able to identify the use of Isoniazid 100mg and use of Isonizaide 300mg and inadequate or insufficient, when knowing the indication for the use of only one of the doses. The attitude will be considered appropriate when in possession of the prescription, the professional checks the presentation of Isoniazid and inappropriate when not checking before dispensing the drug. Regarding the practice, it will be divided into two groups: those who keep the two presentations in different places (group 1) and those who keep the two presentations in the same place (group 2).











Figure 3- timeline for selecting the study sample

Third Stage

It will be a qualitative research that, according to Denzin and Lincoln (2006), involves an interpretive approach to the world, which means that its researchers study things in their natural settings, trying to understand phenomena in terms of the meanings that people give to them. The theoretical axis adopted will be that of social representations, understood as modalities of practical knowledge, oriented towards communication and understanding of the social, material and ideational context in which we live.

The contents expressed by the individual are essentially heterogeneous; they translate the thinking, the common sense of a given social group, understanding the individual in a social perspective, because reality is socially constructed (SPINK, 2004). For Marx (1994) the human essence is not something abstract and immanent to each individual. It is, a reality, built in the set of social relationships that can be reported, can be observed; the speeches and experiences of social actors provide relevant data for the researcher, through meetings and conversations, to expand access to the production of meaning and the representations of individuals constructed (SPINK, 2004).

As a method, the collective subject discourse (CSD) will be used, a methodological strategy in qualitative research for the construction of the CSD, which consists of a qualitative way of representing the thinking of a collectivity, aggregating, in a synthesis discourse, the discursive contents of similar meaning issued by different people. Thus, each individual interviewed in the study, chosen based on criteria of social representativeness, contributes with his share of a fragment of thought for collective thinking (LEFÈVRE and LEFÈVRE, 2004). This methodological procedure implies a definition, based on an empirical perspective, that the collective character of social thought is the amount of choices made by a certain set of individuals belonging to a certain community; although expressed individually, it is socially shared, translating the nature of collective thinking (LEFÈVRE and LEFÈVRE, 2004).

Based on the theory of social representation, the CSD is a method of presenting qualitative research results that includes testimonies as raw material in the form of one or several synthesis discourses, written in the first person singular, an expedient that aims to express the thought of a collectivity, as if this collectivity were the issuer of a discourse (LEFRÈVE, CRESTANA and CORNETTA, 2003). It is the sharing of ideas and positions. A collective mirror; the speech of a social group.

**RESEARCH PARTICIPANTS**

The research participants will be health professionals from primary care and tuberculosis reference centers. The criteria for participating in the research correspond to: over 18 years of age and having at least six months of service for health professionals. Exclusion criteria: declaring not wanting to be a participant in the research, being an intern or having less than six months of effective service.

**DATA COLLECTION INSTRUMENT**

For data collection, a semi-structured interview will be used (APPENDIX B). When relating discursive practices with the production of meaning, it is assumed that the meanings are not in language as materiality, but in the discourse that makes language the tool to build reality (SPINK, 2004). The interview materializes, through language, this individual's experience, which is, for Shotter, a psychological instrument or tool through which we establish different relationships with those around us and produce meaning for our circumstances, enabling the communication of this meaning (apud PINHEIRO , 2004).

The interview will consist of two parts: one for the identification of the interviewees and another for the guiding questions. The formulation of the questions will be natural, it is often not interesting to ask a direct question, but to lead the respondent to remember part of his life. For that, the researcher can very well provoke his memory (BOURDIEU, 2002).

The guiding questions will be separated for the two focus groups and described below:

For Primary Care professionals:

- What do you know about latent tuberculosis infection (LTBI)?

- How do you perform the treatment of LTBI?

- Did you have prior knowledge of Isoniazid 300mg?

- Did you feel safe using Isoniazid 300mg?*

- Was the information material available on Isoniazid 300mg sufficient?

- How was the distribution/dispensing flow of Isoniazid 300mg in your workplace?

- Have you noticed any difference in patients using Isoniazid?

300mg?

For Tuberculosis Reference Center Professionals:

- Did you have prior knowledge of Isoniazid 300mg?

- Did you feel safe using Isoniazid 300mg?*

- Was the information material available on Isoniazid 300mg sufficient?

- How was the distribution/dispensing flow of Isoniazid 300mg in your workplace?

- Have you noticed any difference in patients using Isoniazid?

300mg?

*The term usage tries to encompass the entire process (from prescription to dispensing) by all professional categories involved (doctor, nurse, pharmacist, technicians).

The interview, essentially describing the identification of the research participant, will be carried out individually. The data to be collected by the researchers will strictly follow those induced by the guiding questions available in a script. In the interview, the technique of free association will be used which, according to Tura (1997), has been shown to be especially useful in studies of stereotypes, perceptions and attitudes, elements that make up the structure of social representations. Therefore, in the community, respondents will be asked at the beginning what the word tuberculosis reminds them of or suggests.

Lefèvre and Lefèvre (2005a) emphasize that the fact that it is a qualitative research does not allow the interviewer to introduce new questions, however, if there is a need to facilitate the continuity of the testimony during the interview, add phrases such as: " What else?" "Have you anything else to say?" "Like this?" "Because?" "Want to supplement with something else?"

Before starting each interview, the subjects will be informed about the objectives of the study and the importance of the recording, assuring them of the confidentiality of all statements and the freedom to refuse to participate in the research or to suspend it at any time, without prejudice to him who was asked to sign a Free and Informed Consent Term (APPENDIX A).

**DATA ANALYSIS**

Qualitative analysis will be characterized by seeking the meanings in the subjects' speech, linked to the context. After transcribing the interviews, exhaustive readings will be carried out until the entire text is exhausted, separation of the themes worked and adequacy to the objectives of the study (OLIVEIRA, 2008).

The technique basically consists of analyzing the verbal material collected in surveys that have testimonies as their raw material, extracting from each of these testimonies. Data analysis was performed using the collective subject discourse (CSD), a technique developed by Lefèvre and Lefèvre to organize the material resulting from fieldwork, usually from speeches from interviews. The result is a synthesis-discourse, the result of fragments of individual discourses gathered by similarity of meaning. The individual speech reveals the collective speech (LEFÈVRE AND LEFÈVRE, 2006), it is the junction of individual speeches, respecting the senses and the level of sharing.

Methodological figures were used to construct the speeches: key expressions (E-Ch); central ideas (Ucs); the collective subject discourse (CSD) itself:

• The E-Ch are selected excerpts from the verbal material of each statement, which best describe the content;

• The CIs describe the meanings, or linguistic expression that reveals and describes, in a synthetic and precise way, the meaning present in the statements, using the interviewee's words, not constituting interpretation;

• The anchoring process is the manifestation of a theory, ideology or belief that the authors of the discourse profess and believe that it brings the basic idea that sustains the discourse (LEFÈVRE AND LEFÈVRE, 2005).

If contradictory statements appear regarding the same issue, the CSDs are prepared for the concordant and discordant statements; As the CSDs emerge and the system of interpretation of reality is built and relationships are established in the social context, behaviors and practices become evident, being enriched with social representations that can contribute to the understanding of health care (DUARTE, MAMEDE , ANDRADE, 2009).

The Collective Subjects' Discourses are the gathering of the E-CH present in the testimonies, which have CIs with a similar or complementary meaning, to give them the form of chained sentences. It is important to point out that the CI is not an interpretation, but a description of the meaning of a statement or a set of statements. The technique for building the DSC involves selecting, from each individual answer to a question, the E-CHs, which are the most significant parts of these answers. These E-CH correspond to ICs, which are the synthesis of the discursive content manifested in the E-CH. With the material of the E-CHs of similar CIs, synthesis discourses, or CSDs, are constructed in the first person singular, with a collectivity appearing as if it were an individual discourse. (LEFÈVRE; LEFÈVRE, 2005b).

The CSD represents the thinking of a collectivity, through a series of testimonies that culminate in synthesis discourses, which bring together responses from different individuals, with discursive content of similar meaning, which begins to express or represent social speech or collective thinking. in the first person singular (LEFÈVRE; LEFÈVRE, 2005b).

According to Lefèvre and Lefèvre (2005b), the “I” of the CSD is an attempt to rescue the “social or collective self”; more precisely, it is a resource created to bring out the social unconscious that speaks in the individual. Therefore, the CSD is a discourse to signal what society imposes on the individual through the unconscious internalization of their discourses, which is why it needs to be stated in the first person singular. The speeches reveal what the community thinks, how it thinks, and how this thought is distributed in the social space (LEFÈVRE; LEFÈVRE and MARQUES, 2009).

Subjects will be identified with the capital "C" consonant, followed by the Arabic number increasing from 1. Example: C1; C2; C3; C4. Duarte, MAMEDE & ANDRADE (2002) propose that the complete transcripts of the recorded testimonies be attached to the research, so that they can contribute to the reliability and legitimacy of the results. Even so, it was decided not to put this material in an appendix, as it was considered that reading the individual speeches in full could compromise the confidentiality commitment assumed with the interviewees.

# SCHEDULE

The research project will be carried out according to the following schedule:

| Stages | Duration |
| --- | --- |
| Bibliographic Review | May/2018 to February/2019 |
| Submission of the research project to CEP- CCS-UFES | April/2018 |
| Submission of the research project to the local ethics committees or municipal health departments (Brasília, Curitiba, Florianópolis, Ribeirão Preto and Vitória) | May/2018 |
| Data collection from the pragmatic trial (1st stage): Period of insertion of participants | June/2018 to March/2019 |
| Face-to-face meeting in Brasilia with all coordinators | June/2018 |
| Conducting the Cross-sectional Study | June/2018 |
| Analysis of data from the Cross-sectional Study | August/2018 |
| Delivery of the Transversal final product of the study | October/2018 |
| Conducting the qualitative study | September/2018 |
| Analysis of qualitative study data | October/2018 to December/2018 |
| Face-to-face meeting in Brasilia with all coordinators | December/2018 |
| Delivery of the qualitative final product of the study | March/2019 |
| Closing of follow-up of patients included in the Pragmatic Trial | November / 2019 |
| Analysis of data from the Pragmatic Test | December/2022 |
| Delivery of the final product of the pragmatic test | December/2022 |
| Delivery of the Final Project Report | December/2022 |

# BUDGET

| **Items** | **Quantity** | **Unit Value** | **Total Value** |
| --- | --- | --- | --- |
| COST |  |  |  |
| Daily | BRL 224.00 (BSB)  BRL 177.00 (Ribeirão Preto)  BRL 200.60 (CWB and FLP) | Daily allowances for study trips. Capital trip 2 days and meeting trip 1 day Value based on the Federal Government's daily rate table | BRL 13,460.20 |
| Airline tickets | R$ 700.00 | Airfare for coordinators and facilitators per segment. 2 trips to each capital (R$ 700.00 x 8 trips x 2 people) and 2  team (R$ 700.00 x 10 people) | BRL 18,200.00 |
| Material of  Consumption |  |  | BRL 59,777.30 |
| Post Office |  |  | BRL 12,000.00 |
| Research grant |  |  | BRL 135,450.00 |
| Legal Entity |  |  | BRL 101,059.38 |
| CAPITAL |  |  |  |
| Equipment |  |  | BRL 10,000.00 |
| Total |  |  | 349,946.88 |

# EXPECTED RESULTS

The study intends to support theoretical and operational strategies that respond to the demand to incorporate a new presentation of Isoniazid for the treatment of LTBI in the SUS network by the PNCT/MS that does not add risks to patients. In this sense, it is intended to identify elements of the care process from the prescription to the dispensing and taking of the medication by the individual.

# BIBLIOGRAPHIC REFERENCES

BOURDIEU, P. A ilusão biográfica. In: AMADO J. FERREIRA, M. M. **Usos e abusos da história oral.** 5. ed. Rio de Janeiro: Fundação Getúlio Vargas, 2002.

Christensen E. Methodology of superiority vs. equivalence trials and non-inferiority trials. J Hepatol, v.46, n.5: 947-54, 2007.

DAHER, André et al . Using a single tablet daily to treat latent tuberculosis infection in Brazil: bioequivalence of two different isoniazid formulations (300 mg and 100 mg) demonstrated by a sensitive and rapid high-performance liquid chromatography-tandem mass spectrometry method in a randomised, crossover study. **Mem. Inst. Oswaldo Cruz**, Rio de Janeiro , v. 110, n. 4, p. 543-550, June 2015 . Available from

<<http://www.scielo.br/scielo.php?script=sci_arttext&pid=S0074-> 02762015000400543&lng=en&nrm=iso>. access on 26 Mar. 2018. Epub June 02,

2015. [http://dx.doi.org/10.1590/0074-02760140458.](http://dx.doi.org/10.1590/0074-02760140458)

DENZIN, N. K. e LINCOLN, Y. S. Introdução: a disciplina e a prática da pesquisa qualitativa. In: DENZIN, N. K. e LINCOLN, Y. S. (Orgs.). O planejamento da pesquisa qualitativa: teorias e abordagens. 2. ed. Porto Alegre: Artmed, 2006. p. 15-41 DUARTE, S. J. H.; MAMEDE, M. V.; DE ANDRADE, S. M. Oliveira. Opções

Teórico-Metodológicas em Pesquisas Qualitativas: Representações Sociais e Discurso do Sujeito Coletivo. **Saúde Soc**. São Paulo, v.18, n.4, 2009. p. 620-626.

Kaliyaperumal, K**.**Guideline for Conducting a Knowledge, Attitude and Practice (KAP) Study. Community Ophthalmology, v.4, n.1: p. 7-9, 2004.

LEFEVRE, Ana. Maria. Cavalcanti. CRESTANA, Maria Fazanelli ; CORNETTA, Vitória Kedy. A utilização da metodologia do discurso do sujeito coletivo na avaliação qualitativa dos cursos de especialização “Capacitação e Desenvolvimento de Recursos Humanos em Saúde-CADRHU”, São Paulo - 2002 **Saúde e Sociedade** v.12, n.2, p. 68- 75, jul./dez. 2003.

LEFEVRE, Fernando; LEFEVRE, Ana. Maria.Cavalcanti.**Depoimentos e discursos.Uma proposta de análise em pesquisa social.** Brasília: Liber Livro Editora, 2005.

.; .. **O *discurso do sujeito coletivo*:** um novo enfoque em pesquisa qualitativa (desdobramentos). 2. ed. Caxias do Sul: Educs, 2005.

; .O sujeito coletivo que fala. Interface. **Comunicação, Saúde e Educação**. v. 10, n. 20, 2006. 517-524.

LEFÈVRE Fernando, LEFÈVRE Ana. Maria.Cavalcanti. O pensamento coletivo como soma qualitativa. 2004 [acessado 2016 abr 26]. Disponível em: [http://hygeia.fsp.usp.br/~flefevrel.](http://hygeia.fsp.usp.br/~flefevrel)

LEFEVRE, Fernando; LEFEVRE, Ana Maria Cavalcanti; MARQUES, Maria Cristina da Costa. Discurso do sujeito coletivo, complexidade e auto-organização. Ciênc. saúde coletiva, Rio de Janeiro ,v. 14, n. 4, p. 1193-1204,Aug.2009 .Available from

<<http://www.scielo.br/scielo.php?script=sci_arttext&pid=S1413-> 81232009000400025&lng=en&nrm=iso>. access on27maio.2016.

Matilde Sanchez M, Chen X. Choosing the analysis of population in non-inferiority studies: per protocol or intent-to-treat. Stat Med, v.25. n. 7: p.1169-81, 2006.

MARX, K. O Capital. 14.ed. São Paulo: Difel, 1994. v.1.

MOSCOVICI, Serge. Representações sociais: investigações em psicologia social. Petrópolis: Vozes, 2003.

REHAL, S. et al. Non-inferiority trials: are they inferior? A systematic review of reporting in major medical journals. BMJ Open, 6:e012594, 20. 16. Disponível em:

<[http://bmjopen.bmj.com/content/bmjopen/6/10/e012594.full.pdf.](http://bmjopen.bmj.com/content/bmjopen/6/10/e012594.full.pdf) Acesso em 20 de março de 2018.

SCHULZ, K. F.; ALTMAM, D. G., MOHEN, D. CONSORT 2010 Statement: updated

guidelines for reporting parallel group randomised trials. BMJ, V. 130, 2010. Disponível em:

<<http://www.consortstatement.org/Media/Default/Downloads/CONSORT%202010%20> Statement/CONSORT%202010%20Statement%20(BMJ).pdf. Acesso em: 20 de março de 2018.

SPINK, M. J. (Org.). **Práticas discursivas e produção de sentidos no cotidiano:**

aproximação teórica e metodológicas**.** 3.ed. São Paulo: Cortez, 2004.

TURA, L. F. R. Os Jovens e a Prevenção da AIDS no Rio de Janeiro. 1997. 174 p. Tese Janeiro, Rio de Janeiro.WHO.World Health Organization. 2009. **Global tuberculosiscontrol**: epidemiology, strategy, financing. Geneva, WHO Report. Geneva, Switzerland, 2009. ([http://www.who.int/gho/tb/epidemic/cases_deaths/en/index.html:](http://www.who.int/gho/tb/epidemic/cases_deaths/en/index.html)

Acesso em 22 Junho de 2016).

ASSOCIAÇÃO BRASILEIRA DE NORMAS TÉCNICAS. **NBR ISSO/IEC 25062:**

**2011:** Engenharia de software — Requisitos e avaliação da qualidade de produto de software (SQuaRE) — Formato comum da indústria (FCI) para relatórios de teste de usabilidade.

BRANCO, M. A. F. Sistemas de informação em saúde no nível local. **Cad. Saúde Pública**, Rio de Janeiro , v. 12, n. 2, jun. 1996.

BRASIL. Ministério da Saúde. Gabinete do Ministro. PORTARIA Nº 2.073, de 31 de Agosto de 2011. Regulamenta o uso de padrões de interoperabilidade e informação em saúde para sistemas de informação em saúde no âmbito do Sistema Único de Saúde, nos níveis Municipal, Distrital, Estadual e Federal, e para os sistemas privados e do setor de saúde suplementar. **Diário Oficial da União**, Brasília, 31 de agosto de 2011c.

FEIJÓ, V.C.; GONÇALVES, B.S.; GOMEZ, L.S.R. Heurística para avaliação de usabilidade em interfaces de aplicativos smartphones: utilidade, produtividade e imersão. **Design & Tecnologia**., v. 3, n. 6, p. 33-42, 2013 Sup:1.

GERMAN, R. R. et al. **Guidelines Working Group Centers for Disease Control and Prevention (CDC).** Updated guidelines for evaluating public health surveillance systems: recommendations from the Guidelines Working Group*.* MMWR Recommendations and Reports, v. 50, n. RR-13, Jul 27, p. 1-35; quiz CE1-7, 2001.

GOOGLE. **Android Studio**. Disponível em:

<https://developer.android.com/studio/intro/index.html?hl=pt-br>. Acesso em: 18 out. 2017.

HERBSLEB, James D. **Global software engineering**: The future of socio-technical coordination. In: 2007 Future of Software Engineering. IEEE Computer Society, p. 188- 198, 2007.

KNOLL RC. **Desenvolvimento de heurísticas de usabilidade para tablets**. [Dissertação]. Florianópolis(SC): Universidade Federal de Santa Catarina; 2014.

Kraut R. Unesco policy guidelines for mobile learning. United Nations Educational, Scientific and Cultural Organization (UNESCO) [Internet]. Paris (FR); 2013. [Acesso

17 set 2017]. Disponível em: [http://unesdoc.unesco.org/images/0021/002196/219641E.pdf.](http://unesdoc.unesco.org/images/0021/002196/219641E.pdf)

LEWIS, J. R. IBM computer usability satisfaction questionnaires: Psychometric evaluation and instructions for use. **Int. J. Hum. Comput. Interact**., v. 7, n. 1, p. 57– 78, Jan. 1995.

LEITE JÚNIOR, J. C., RAMOS, R. T. T., ROBAZZi, T. C. M. V. Tratamento da

tuberculose latente em pacientes com doenc¸as reumáticas juvenis: uma revisão sistemática. **Rev Bras Reumatol**, v. 57, n. 3, p. 245-253, 2017.

LEWIS, J. R. Usability: Lessons Learned ... and Yet to Be Learned. **Int. J. Hum. Comput. Interaction**, v. 30, n. 9, p. 663-684, 2014.

MICROSOFT. **Language Understanding Intelligent Service (LUIS)**. Disponível em:

<https:/[/www.luis.ai/hom](http://www.luis.ai/home)e>. Acesso em: 18 out. 2017.

MINAYO, M. C. S. **O desafio do conhecimento**. São Paulo: Hucitec, 1993.

NIELSEN, J. Conference companion on human factors in computing systems. In: ACM. Proceedings... [S.l.], 1994. p. 413–414. Acesso em: maio 2015.

ORACLE. **Java**. Disponível em: <https:/[/www.java.com/pt_B](http://www.java.com/pt_BR/)R[/>.](http://www.java.com/pt_BR/) Acesso em: 18 out. 2017a.

ORACLE. **MySQL**. Disponível em: <https://[www.mysql.com/](http://www.mysql.com/)>. Acesso em: 18 out. 2017b.

PRESSMAN, R. **Software engineering: a practitioner's approach.** 7 ed. Porto Alegre: McGraw-Hill, 2010. 895p.

REDDY, M.; PRATT, W.; DOURISH, P.; et al. Sociotechnical requirements analysis for clinical systems. **Methods of Information in Medicine**, v. 42, p. 437-444, 2003.

RIJO RPCL; ALVES, D. Software Evaluation From the Perspective of Patients and Healthcare Professionals. **Encyclopedia of Information Science and Technology**, Fourth Edition. 4ed.: IGI Global, p. 3782-3793, 2017.

RIJO, R.P.C.L., CREPALDI, N.Y., BERGAMINI, F. et al. Impact assessment on patients satisfaction and healthcare professionals commitment of software supporting Directly Observed Treatment, Short-course: A protocol proposal. **Health Informatics Journal**, v. 23, p. 146045821771205, 2017.

SALAZAR, L. H. A.; LACERDA, T. C.; NUNES, J. V.; WANGENHEIM, C. G. von.

Systematic Literature Review on Usability Heuristics for Mobile Phones. International Journal of Mobile Human Computer Interaction, v. 5, n. 2, 2013.

SOMMERVILLE, I. **Engenharia de software**. 9 ed. São Paulo: Pearson, 2011. 552p.

TIBES, C.M. Aplicativo Móvel para prevenção e classificação de Úlceras por pressão. 2014. 118 f. [dissertação]. Programa de Pós-Graduação em Enfermagem, Universidade Federal de São Carlos; 2015

THE PHP GROUP. **PHP: Hypertext Preprocessor**. Disponível em:

<https://secure.php.net/manual/pt_BR/intro-whatis.php>. Acesso em: 18 out. 2017.

VESCOVI, S. de J. B. et al . Aplicativo móvel para avaliação dos pés de pessoas com diabetes mellitus. **Acta paul. enferm.**, v. 30, n. 6, p. 607-613, Dec. 2017 .

VINCI, A.; RIJO, R.; AZEVEDO-MARQUES, J.; ALVES, D.. Proposal of an

evaluation model for mental health care networks using information technologies for its management. **International Conference on Health and Social Care Information Systems and Technologies HCist ‘2016**. Porto 5-7 October 2016.

WORLD WIDE WEB CONSORTIUM - W3C. **eXtensible Markup Language XML**. Disponível em: <https://[www.w3.org/XML/>.](http://www.w3.org/XML/) Acesso em: 18 out. 2017.

YOSHIURA, V.; AZEVEDO-MARQUES, J.; ALVES, D.. Proposal to develop a web- based observatory as a management, research and assistance tool for a public mental health care network. **International Conference on Health and Social Care Information Systems and Technologies HCist ‘2016**. Porto 5-7 October 2016.

YOSHIURA, V. T., DE AZEVEDO-MARQUES, J. M., RZEWUSKA, M., VINCI, A.

L. T., SASSO, A. M., MIYOSHI, N. S. B. ALVES, D. (2017). A web-based information system for a regional public mental healthcare service network in Brazil. **International Journal of Mental Health Systems**, v.11, n.1, 2017.

# APPENDICES

APPENDIX 1:

Informed Consent Term (TCLE) - 1st Step

Resolution No. 466/2012 - National Health Council

Mr. (a) ____________________________________________, you were invited to participate in a stage of the research entitled Evaluation of the implementation of isoniazid 300mg for the treatment of Latent Tuberculosis Infection under the responsibility of Prof. Dr Ethel Leonor Noia Maciel.

RESEARCH OBJECTIVE: This stage of the research will evaluate the use of two available doses of isoniazid (100mg and 300mg) for the treatment of latent tuberculosis.

PROCEDURES: Your participation will take place by signing this term in two copies (one for the participant and the other for the researcher). In this study we will evaluate the new formulation of isoniazid 300 mg. So, instead of taking 3 (three) pills of 100mg you can take 1 pill of 300mg.

In addition, you will be interviewed at 4 (four) time points (week 2 of treatment, first month, second month, and end of treatment). The collected data will only be used IN THIS research and the results published in events and/or scientific journals.

DURATION AND RESEARCH SITE: 9 to 12 months in the Health Facility responsible for the treatment.

RISKS AND DISCOMFORTS: The risks are related to the adverse effects of the medication. However, the medication evaluated in this study is approved by ANVISA (National Health Surveillance Agency) and has already been used by TB control programs in the treatment of latent TB. Therefore, in this study we will evaluate the new formulation of isoniazid 300 mg, that is, instead of taking 3 (three) 100mg tablets you will take 1 300mg tablet. If you feel any discomfort, you should look for the health service and the project researchers.

BENEFITS: We believe that with the development of this project it will be possible to insert a new scheme for the treatment of latent TB. Therefore, it will contribute to the prevention and control of Tuberculosis.

GUARANTEE OF REFUSAL TO PARTICIPATE IN THE RESEARCH: Mr. (a) is not

obliged to participate in the research, being able to stop participating in it at any time of its execution, without any penalties or damages resulting from its refusal. If you decide to withdraw your consent, Mr. (a) will no longer be contacted (a) by researchers.

GUARANTEE OF MAINTENANCE OF CONFIDENTIALITY AND PRIVACY:

confidentiality of all data obtained. Each participant will be identified only by a participation number, known only to the researchers. No results will be reported with personal identification. All care will be taken to maintain the non-identification of the participant. The collected data will be entered in the research results, which will be retained by the researcher, for use of this information in the work, and can be used for dissemination in national and international newspapers and/or scientific journals. If the research is published, any and all identities will remain confidential.

FINANCIAL REIMBURSEMENT AND/OR INDEMNITY GUARANTEE: A

research will have no cost or any financial compensation. QUESTIONS: In case of questions about the research or the need to report any injury or damage related to the study, I should contact the researcher Ethel Leonor Noia Maciel, on the phone (27) 999733123 or e-mail ethel.maciel @gmail.com. If you are unable to contact the researcher or to report a problem, you can also contact the CCS/UFES Research Ethics Committee at (27) 3335-7211, e-mail cep.ufes@ hotmail.com or mail, through the following address: Federal University of Espírito Santo, Ethics Committee in Research with Human Beings, Av. Marechal Campos, 1468 – Maruípe, CCS Administration Building, CEP 29.040-090, Vitória - ES, Brazil. The CEP/CCS/UFES is responsible for analyzing research projects aimed at protecting participants within national and international ethical standards. Its opening hours are from Monday, from 8:00h to 12:00h and from 13:00h to 17:00h.

I ______________________________________________declare that I have been verbally informed and clarified about the content of this document, understanding all the above terms, as well as my rights, and that I voluntarily agree to participate in this study. I also declare that I have received a copy of this Informed Consent Term, of equal content, signed by the researcher and initialed all pages.

Espírito Santo,____________________________________________________

RESEARCH PARTICIPANT

As a researcher in the study “Evaluation of the implementation of isoniazid 300mg for the treatment of Latent Tuberculosis Infection”, I _______________________________ , I declare that I have complied with the requirements of item IV.3 of Resolution CNS 466/12, which establishes guidelines and regulatory standards for research involving human beings.

SIGNATURE OF THE RESEARCHER WHO COLLECTED THE DATA
